# Supplementary material for: The link between steady-state EEG and rs-fMRI metrics in healthy young adults: The effect of macrovascular correction
Source: Imaging Neurosci (Camb). 2026 Jan 12;4:IMAG.a.1092. doi: 10.1162/IMAG.a.1092 (PMC12797145; doi:10.1162/IMAG.a.1092)
Supplement: Supplementary Material [file IMAG.a.1092_supp.pdf]

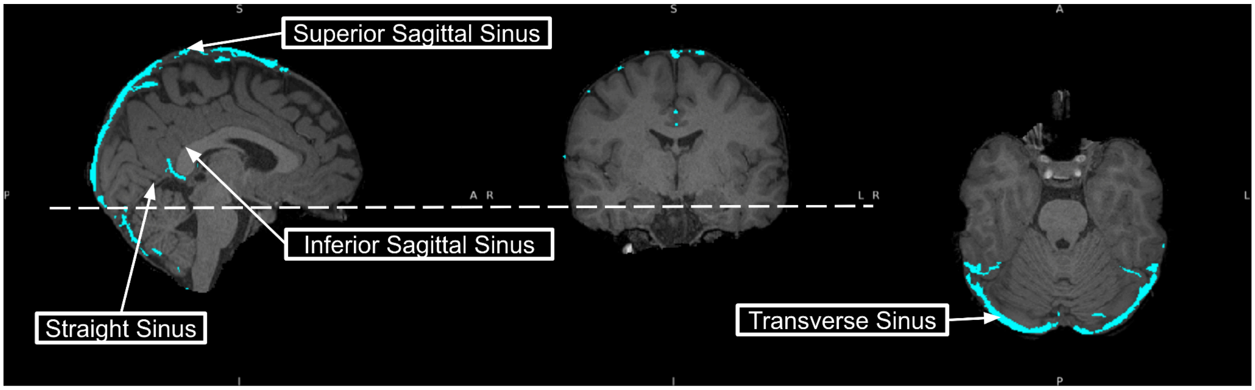

Figure S1. Venous vasculature demonstration overlays the anatomical images and defines the macrovasculature in this study (data shown from a representative data set). Blue indicates the vein segmented from the TOF data overlay on the T1 anatomical image; the white dashed line indicates the axial slice position.

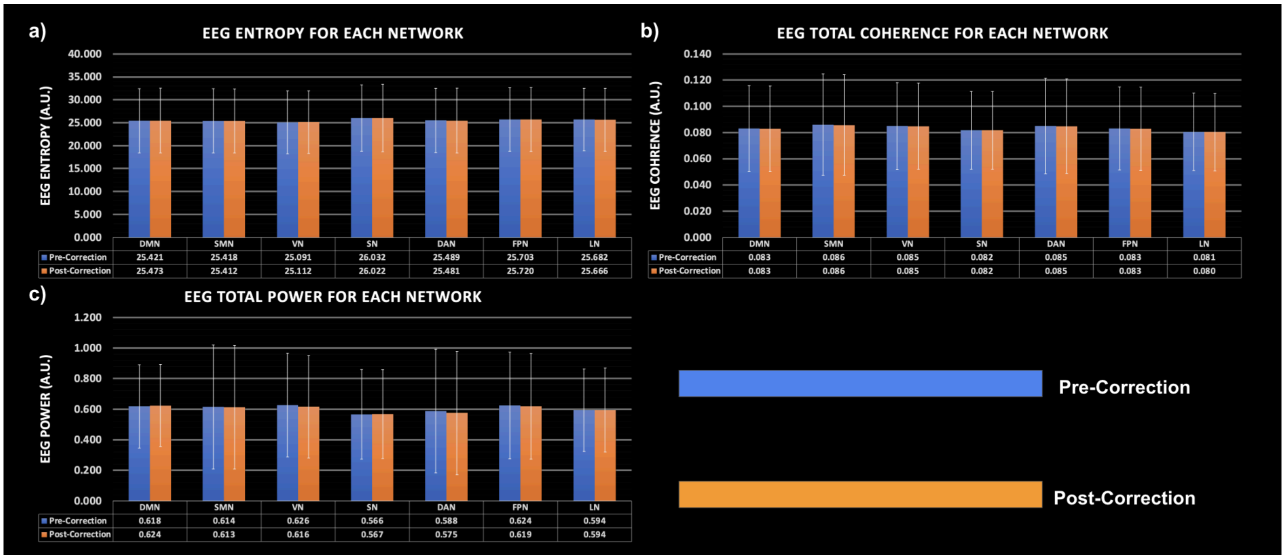

Figure S2. The average EEG band-free metrics using network ROIs are defined based on rs-fMRI data pre- and post-macrovascular correction. The EEG metrics corresponding to the post-correction ROIs are of course, corrected for macrovascular contributions. Grouped bars from left to right: DMN, SMN, VN, SN, DAN, FPN, and LN. Blue: pre-macrovascular correction; orange: post-macrovascular correction. Error bars represent the standard deviation.

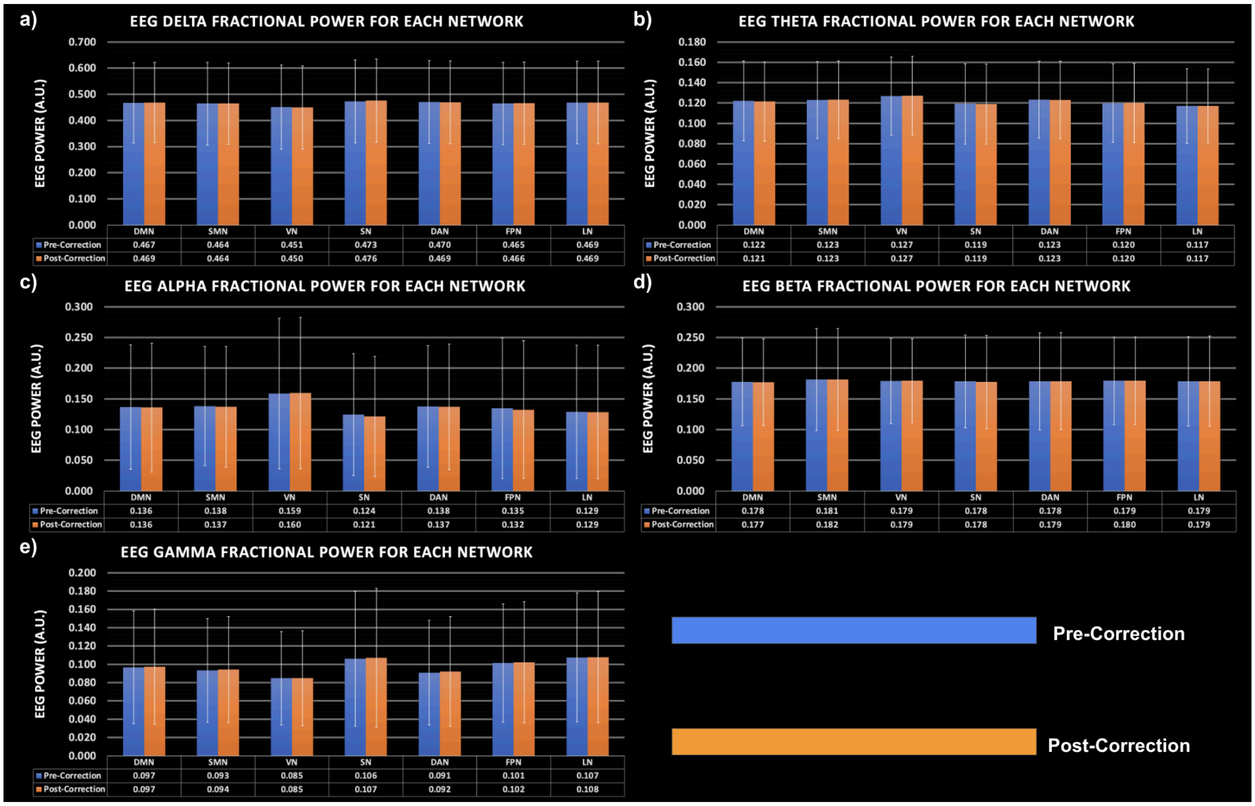

Figure S3. The average EEG band-limited fractional power using network ROIs is defined based on rs-fMRI data pre- and post-macrovascular correction. The EEG metrics corresponding to the post-correction ROIs are of course, corrected for macrovascular contributions. Grouped bars from left to right: DMN, SMN, VN, SN, DAN, FPN, and LN. Blue: pre-macrovascular correction; orange: post-macrovascular correction. Error bars represent standard deviation.

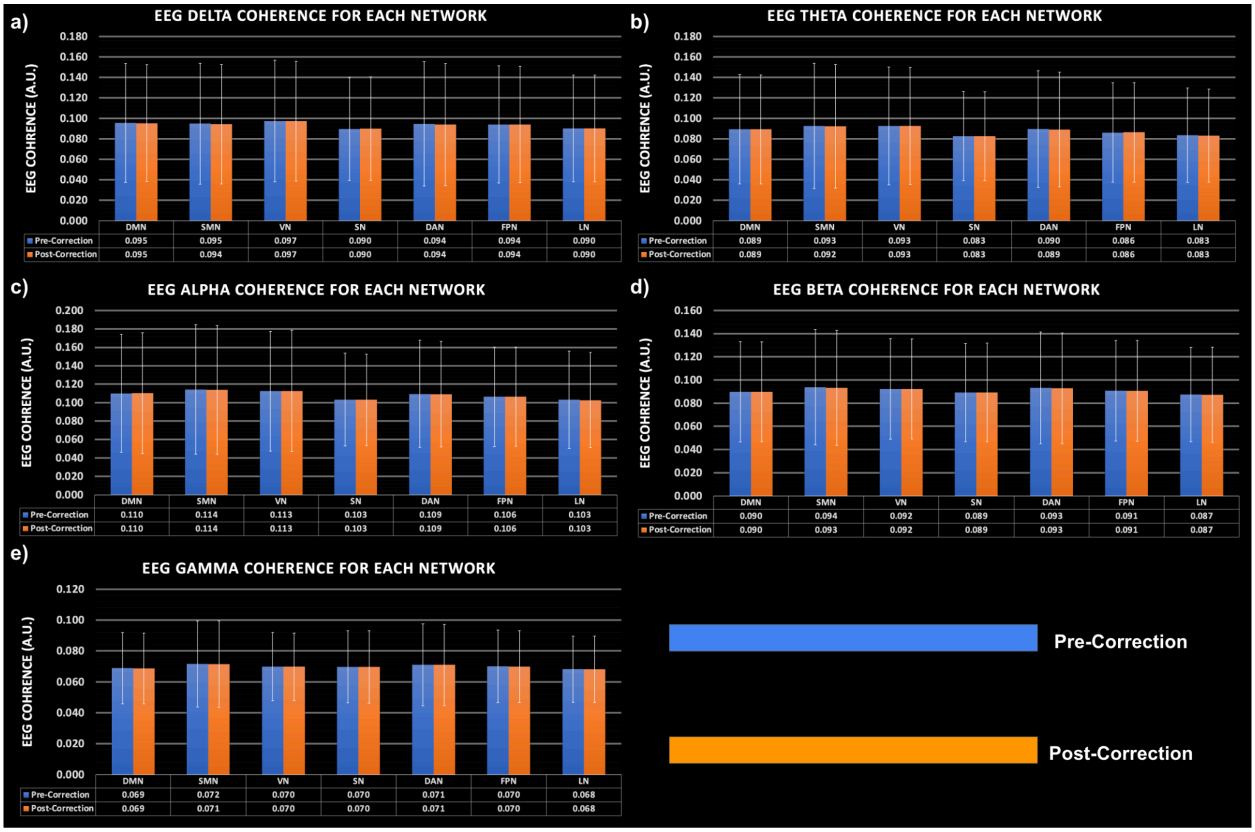

Figure S4. The average EEG band-limited connectivity (coherence) from network ROIs, computed based on rs-fMRI data pre- and post-macrovascular correction. The EEG metrics corresponding to the post-correction ROIs are of course, corrected for macrovascular contributions. Grouped bars from left to right: DMN, SMN, VN, SN, DAN, FPN, and LN. Blue: pre-macrovascular correction; orange: post-macrovascular correction. Error bars represent standard deviation.

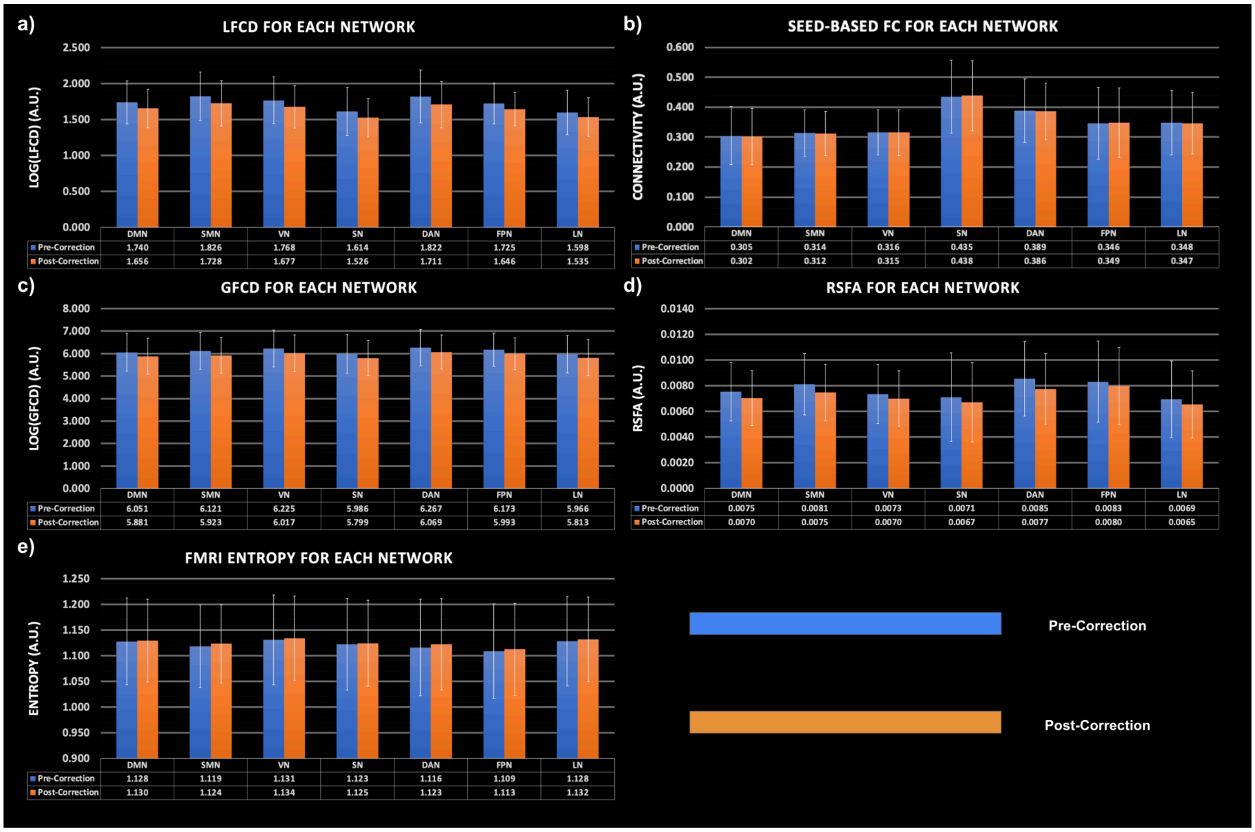

Figure S5. The average rs-fMRI metrics using network ROIs are defined based on rs-fMRI data pre- and post-macrovascular correction. The rs-fMRI metrics corresponding to the post-correction ROIs are of course corrected for macrovascular contributions. Grouped bars from left to right: DMN, SMN, VN, SN, DAN, FPN, and LN. Blue: pre-macrovascular correction and orange: post-macrovascular correction. Error bars represent standard deviation.

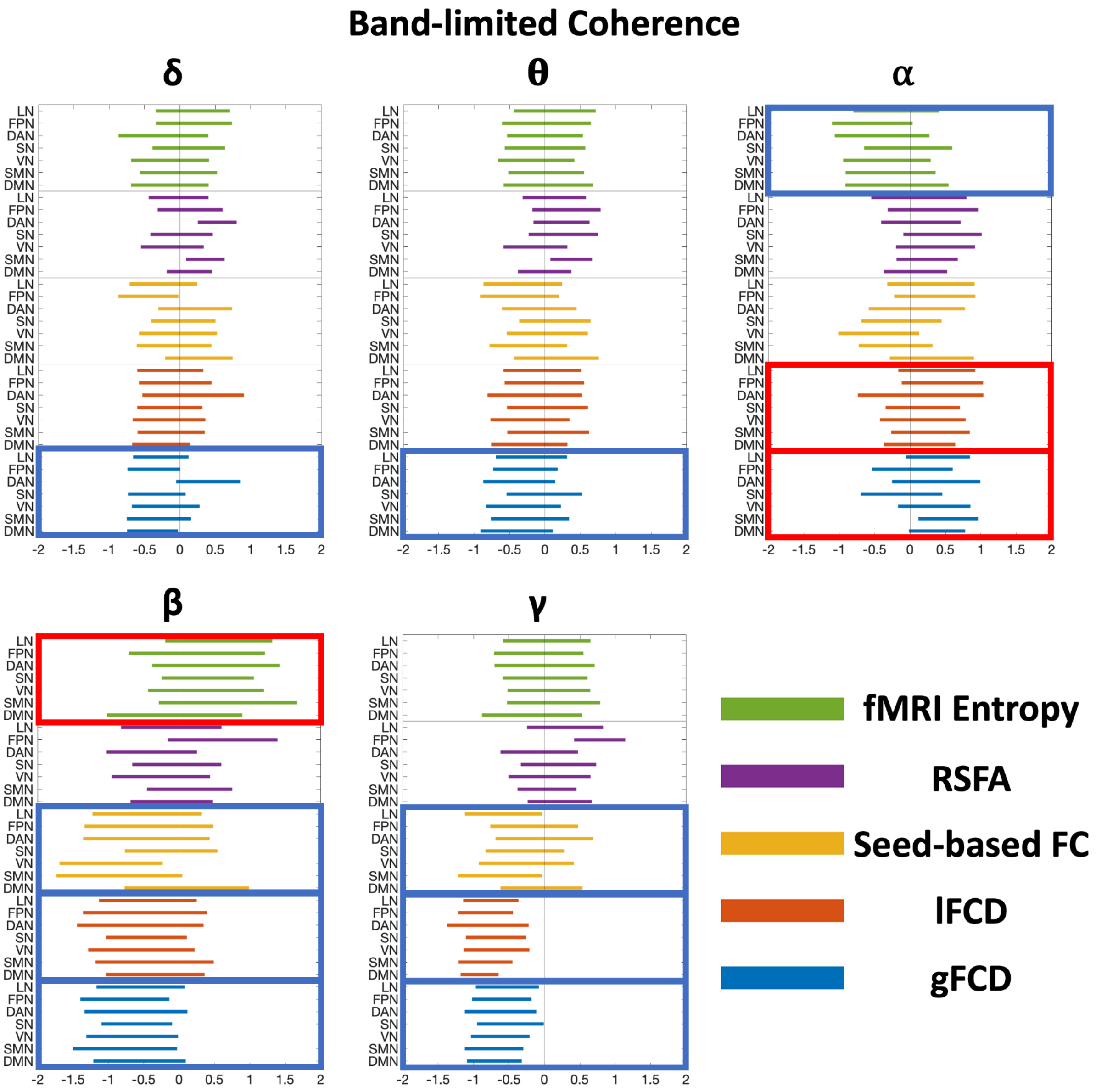

Figure S6. Effect sizes of associations between rs-fMRI metrics and EEG band-limited coherence in each individual network, with macrovascular correction. Each bar represents the 95% confidence interval of the effect size, and its colour corresponds to the rs-fMRI metric as specified by the legend. The boxes indicate significant whole-brain associations with the colour indicating the direction: red for positive and blue for negative.

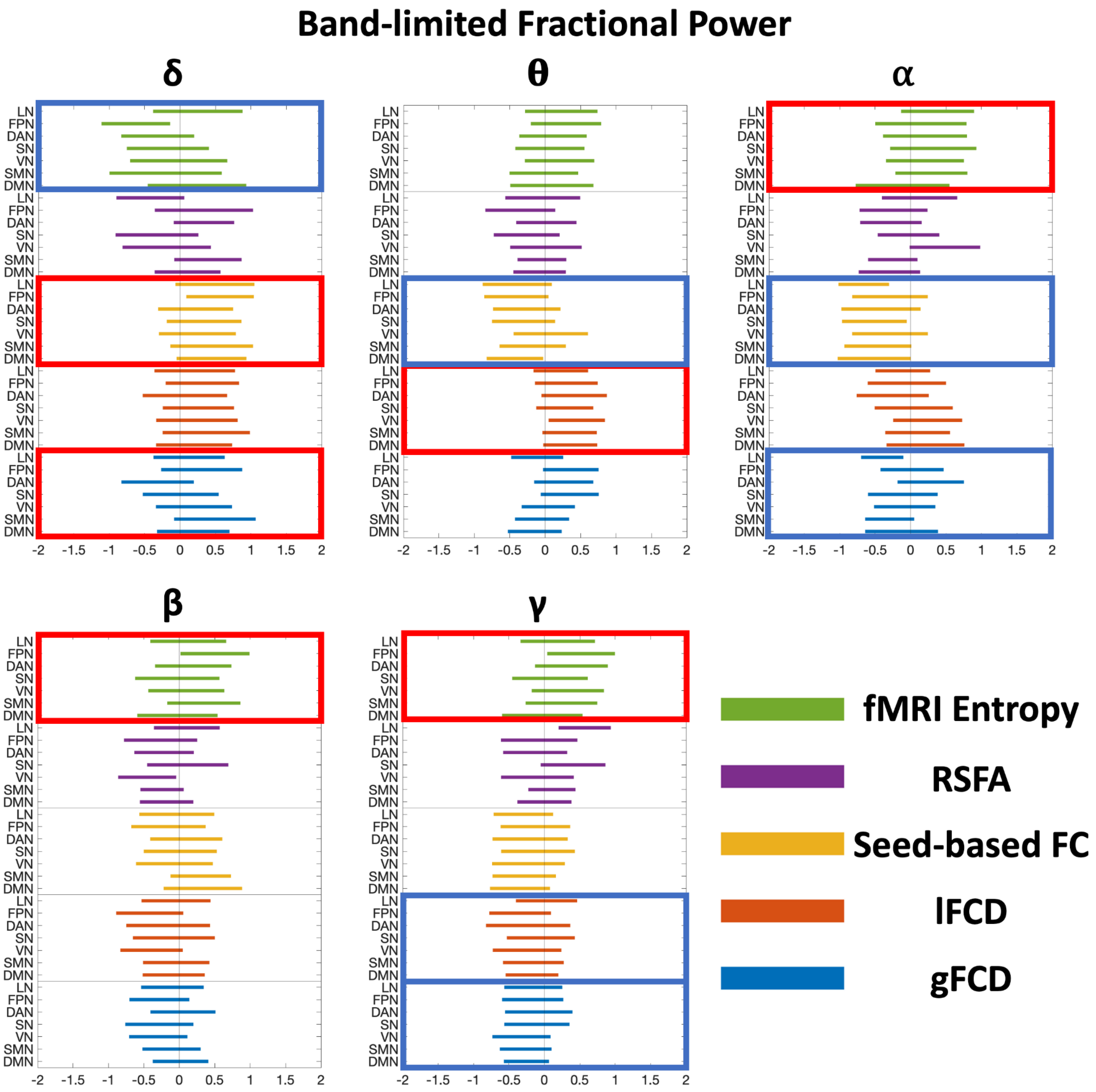

Figure S7. Effect sizes of associations between rs-fMRI metrics and EEG band-limited fractional power in each individual network, with macrovascular correction. Each bar represents the 95% confidence interval of the effect size, and its colour corresponds to the rs-fMRI metric as specified by the legend. The boxes indicate significant whole-brain associations with the colour indicating the direction: red for positive and blue for negative.

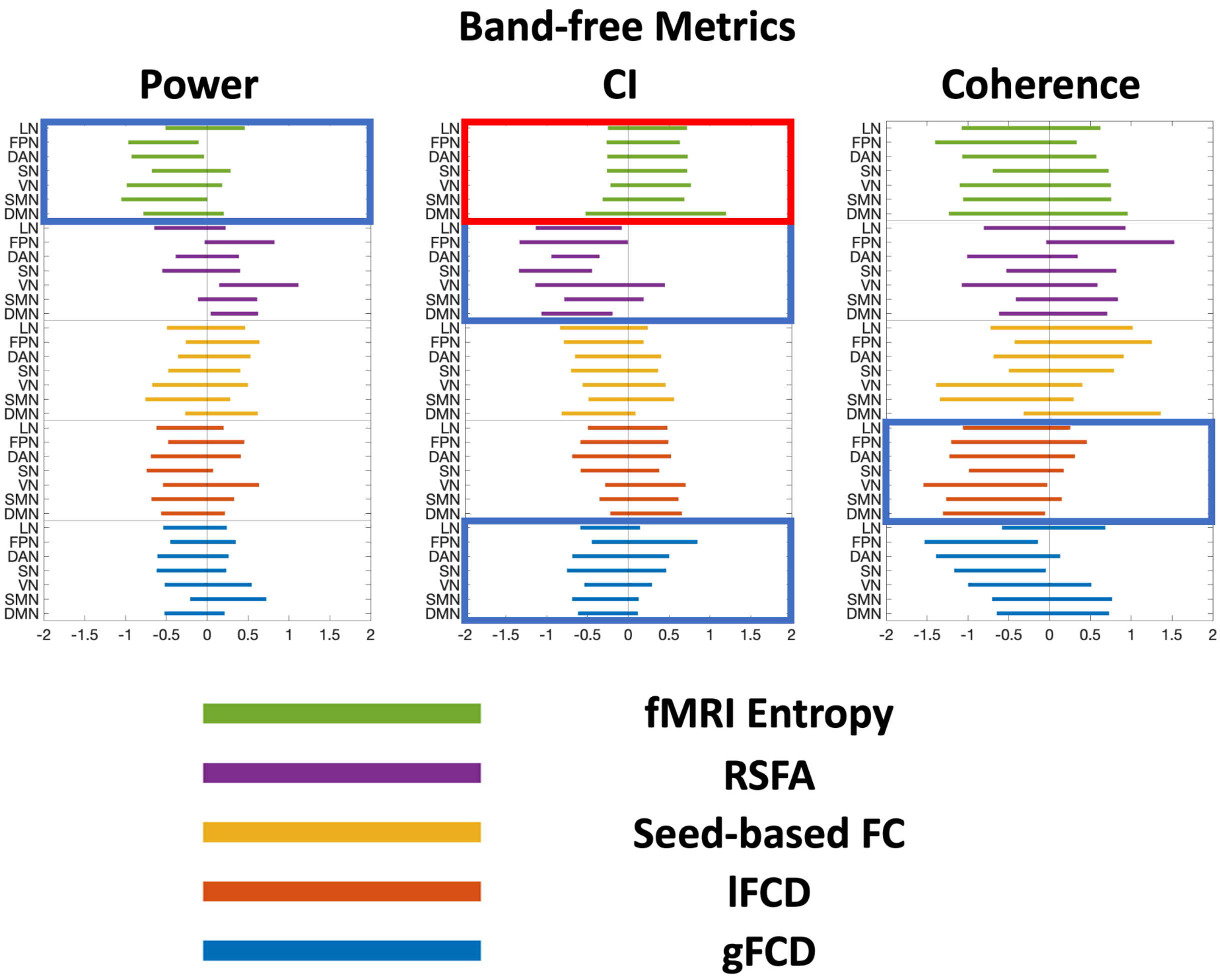

Figure S8. Effect sizes of associations between rs-fMRI metrics and EEG band-free metrics in each individual network, with macrovascular correction. Each bar represents the 95% confidence interval of the effect size, and its colour corresponds to the rs-fMRI metric as specified by the legend. The boxes indicate significant whole-brain associations with the colour indicating the direction: red for positive and blue for negative.

Table S1. Comparison of the effect size from LME models with and without network IDs as random variables (post-correction). LME model with and without network IDs as random variables. Only associations with significant random effects are shown here. There is no noticeable difference between the effect size of significant effects before and after the network IDs are included as a random effect. Even the insignificant effects might go from positive to negative.

	Effect size for EEG variables		Effect size for sex interaction	
	With random effect	Without random effect	With random effect	Without random effect
lFCD vs α Coherence	0.30	0.29	0.29	0.31
lFCD vs γ Coherence	-0.72	-0.71	-0.31	-0.31
Seed-based FC vs δ Coherence	-0.15	-0.15	0.21	0.23
Seed-based FC vs θ Coherence	-0.11	-0.11	0.017	0.023
Seed-based FC vs α Coherence	0.011	0.023	0.37	0.35
Seed-based FC vs β Coherence	-0.39	-0.38	0.056	-0.039
Seed-based FC vs γ Coherence	-0.28	-0.27	0.048	-0.050
lFCD vs θ Power	0.30	0.31	-0.15	-0.15
Seed-based FC vs δ Power	0.31	0.32	0.25	0.26
Seed-based FC vs θ Power	-0.27	-0.28	0.049	-0.055
Seed-based FC vs β Power	0.037	0.029	-0.25	-0.26
Seed-based FC vs γ Power	-0.17	-0.15	-0.15	-0.16
RSFA vs γ Power	0.11	0.075	0.026	0.038
Seed-based FC vs Broadband CI	-0.14	-0.12	-0.14	-0.15
RSFA vs Broadband CI	-0.58	-0.58	-0.18	-0.17
Seed-based FC vs Broadband Power	-0.025	-0.039	0.21	0.24
Seed-based FC vs Broadband Coherence	0.022	0.023	0.30	0.31

Macrovascular segmentation and data processing

The strategies for macrovasculature segmentation and processing are summarised in. TOF images were registered to T1 space (FSL MCFLIRT) and segmented using the Braincharter Toolbox (https://github.com/braincharter/vasculature) (Bernier et al., 2018). Visual inspection was performed to ensure the absence of major artefacts. The centreline image encoded with diameter information (“centredia” output from the Braincharter Toolbox) obtained from the raw images (at 0.7 mm isotropic voxel resolution) were pooled from all TOF images across all encoding directions, and where vessel overlaps were detected, the highest diameter estimate is assumed for the overlapping vessel. Accordingly, the vessels used in the subsequent analysis are primarily major cerebral vessels with a minimum diameter of close to 0.7 mm (for example the superior sagittal sinus and the Circle of Willis). This combinatorial approach maximised the completeness and signal-to-noise ratio of the resulting macro-VANs. The combined centrelines were then upsampled to a resolution of 0.175 mm using AFNI (Cox, 1996) with nearest-neighbour interpolation (3dresample). Based on each vascular centreline, a line was constructed that connects two vascular voxels that both neighbour the central voxel in a 9×9×9 voxel matrix.

Vascular blood-volume fraction (fBV) and orientation maps were manually registered to BOLD space using coordinates from AFNI's volume selection function (3dAutobox). fBV was estimated by counting the number of high-resolution voxels occupied by vessels in each fMRI voxel (4mm isotropic voxel resolution), and macrovascular maps were derived from binarized fBV maps. In order to ensure the accuracy of registration, necessary quality control measures have been added. Due to the fact that all TOF images contained both arteries and veins, each arterial and venous map was manually separated based on an anatomical atlas (Tortora and Derrickson, 2018). Lastly, the measured voxelwise values of orientation and fBV are used to create different simulated voxels containing blood vessels.

Venous signal simulation and correction

A whole-brain susceptibility map was generated numerically using the Fourier method (Eq. 1 and 2), in which a mask of the vasculature was constructed by upsampling macro-VANs to 0.175 mm isotropic resolution and zero-padding by the size of a full field-of-view on each side to avoid wraparounds resulting from cycle convolution (Cheng et al., 2009; Salomir et al., 2003). To calculate the susceptibility difference between blood and tissue outside macrovasculature, we assumed that the tissue type outside macrovasculature is grey matter (GM).

$$\Delta B_z = FT^{-1}[(\frac{1}{3} - \frac{k_z^2}{k^2})FT(\chi)] \tag{1}$$

$$\chi = \Delta\chi \cdot Hct \cdot (1 - Y) \tag{2}$$

FT denotes the Fourier transform, χ the local susceptibility, k_z the distance in the k space along the z-axis and k the distance in k-space ($k^2 = k_x^2 + k_y^2 + k_z^2$). R_2' is then calculated through the

magnitude of the complex-valued mean magnetization of the dephasing spins resulting from the B_0 offset.

The mean BOLD signal was calculated as defined by Eq. 3 and 4.

$$S_{T2'} = |\mu(exp(i\gamma \Delta B_z TE))| \quad (3)$$

$$S = sin(\alpha)(1 - exp(- TR/T1))/(1 - cos(\alpha)exp(- TR/T1)\ exp(- TE/T2))S_{T2'} \quad (4)$$

where γ is the gyromagnetic ratio, and the operator $|\mu(\cdot)|$ represents the magnitude of the mean of a complex number. A summary of the values and definitions used for simulation parameters is provided in **Table S2**.

The simulations were conducted using our servers equipped with 14 cores of Intel Xeon X5687 CPU (at 3.6 GHz) (Intel Corporation, Santa Clara, CA, United States) and 180 GB of memory running Red Hat Enterprise Linux Server 7.7 (Red Hat Inc., Raleigh, NC, United States). A customized simulation script was written in MatLab 2019b (MathWorks Inc., Natick, MA, United States.).

Table S2. Simulation parameters and values. For all three simulation models, these values were set to default unless otherwise stated.

Parameter	Definition	Simulated Value	Source
$\Delta\chi$	Susceptibility of blood with fully deoxygenated blood	$4 \times \pi \times 0.27 \times 10^{-6}$	(Spees et al., 2001)
Hct	Hematocrit	0.4	Men: 40-54%; Women: 36-48% (Billett, 1990)
Voxel size	N/A	3.5 mm isotropic	According to in-vivo rs-fMRI acquisition protocol
TR	Repetition time	4.5 s	
TE	Echo time	30 ms	
α	Flip angle	90 deg	
B_0	Main magnetic field	3T	
Y_v	Venous oxygenation level	0.6	(Fan et al., 2014)
Y_{tissue}	Tissue oxygenation level	0.85	(Gagnon et al., 2015)
$T1_{blood}$	T1 of blood	1649 ms	(Zhang et al., 2013)

$T1_{tissue}$	T1 of tissue	1465 ms	(Shin et al., 2009)

Bernier, M., Cunnane, S.C., Whittingstall, K., 2018. The morphology of the human cerebrovascular system. Hum. Brain Mapp. 39, 4962–4975. https://doi.org/10.1002/hbm.24337

Billett, H.H., 1990. Hemoglobin and Hematocrit, in: Walker, H.K., Hall, W.D., Hurst, J.W. (Eds.), Clinical Methods: The History, Physical, and Laboratory Examinations. Butterworths, Boston.

Cheng, Y.-C.N., Neelavalli, J., Haacke, E.M., 2009. Limitations of calculating field distributions and magnetic susceptibilities in MRI using a Fourier based method. Phys. Med. Biol. 54, 1169–1189. https://doi.org/10.1088/0031-9155/54/5/005

Cox, R.W., 1996. AFNI: software for analysis and visualization of functional magnetic resonance neuroimages. Comput. Biomed. Res. 29, 162–173. https://doi.org/10.1006/cbmr.1996.0014

Fan, A.P., Bilgic, B., Gagnon, L., Witzel, T., Bhat, H., Rosen, B.R., Adalsteinsson, E., 2014. Quantitative oxygenation venography from MRI phase. Magn. Reson. Med. 72, 149–159. https://doi.org/10.1002/mrm.24918

Gagnon, L., Sakadžić, S., Lesage, F., Musacchia, J.J., Lefebvre, J., Fang, Q., Yücel, M.A., Evans, K.C., Mandeville, E.T., Cohen-Adad, J., Polimeni, J.R., Yaseen, M.A., Lo, E.H., Greve, D.N., Buxton, R.B., Dale, A.M., Devor, A., Boas, D.A., 2015. Quantifying the microvascular origin of BOLD-fMRI from first principles with two-photon microscopy and an oxygen-sensitive nanoprobe. J. Neurosci. 35, 3663–3675. https://doi.org/10.1523/JNEUROSCI.3555-14.2015

Salomir, R., de Senneville, B.D., Moonen, C.T.W., 2003. A fast calculation method for magnetic field inhomogeneity due to an arbitrary distribution of bulk susceptibility. Concepts Magn. Reson. 19B, 26–34. https://doi.org/10.1002/cmr.b.10083

Shin, W., Gu, H., Yang, Y., 2009. Fast high-resolution T1 mapping using inversion-recovery Look-Locker echo-planar imaging at steady state: optimization for accuracy and reliability. Magn. Reson. Med. 61, 899–906. https://doi.org/10.1002/mrm.21836

Spees, W.M., Yablonskiy, D.A., Oswood, M.C., Ackerman, J.J., 2001. Water proton MR properties of human blood at 1.5 Tesla: magnetic susceptibility, T(1), T(2), T*(2), and non-Lorentzian signal behavior. Magn. Reson. Med. 45, 533–542. https://doi.org/10.1002/mrm.1072

Tortora, G.J., Derrickson, B.H., 2018. Principles of Anatomy and Physiology. John Wiley & Sons.

Zhang, X., Petersen, E.T., Ghariq, E., De Vis, J.B., Webb, A.G., Teeuwisse, W.M., Hendrikse, J., van Osch, M.J.P., 2013. In vivo blood T(1) measurements at 1.5 T, 3 T, and 7 T. Magn. Reson. Med. 70, 1082–1086. https://doi.org/10.1002/mrm.24550
